# Sustainable batching water options for one-part alkali-activated slag mortar: Sea water and reverse osmosis reject water

**DOI:** 10.1371/journal.pone.0242462

**Published:** 2020-11-13

**Authors:** Tero Luukkonen, Juho Yliniemi, Paivo Kinnunen, Mirja Illikainen

**Affiliations:** Fibre and Particle Engineering Research Unit, University of Oulu, Oulu, Finland; Mirpur University of Science and Technology, PAKISTAN

## Abstract

Concrete production is globally a major water consumer, and in general, drinking-quality water is mixed in the binder. In the present study, simulated sea water and reverse osmosis reject water were used as batching water for one-part (dry-mix) alkali-activated blast furnace slag mortar. Alkali-activated materials are low-CO_2_ alternative binders gaining world-wide acceptance in construction. However, their production requires approximately similar amount of water as regular Portland cement concrete. The results of the present study revealed that the use of saline water did not hinder strength development, increased setting time, and did not affect workability. The salts incorporated in the binder decreased the total porosity of mortar, but they did not form separate phases detectable with X-ray diffraction or scanning electron microscopy. Leaching tests for monolithic materials revealed only minimal leaching. Furthermore, results for crushed mortars (by a standard two-stage leaching test) were within the limits of non-hazardous waste. Thus, the results indicated that high-salinity waters can be used safely in one-part alkali-activated slag to prepare high-strength mortars. Moreover, alkali-activation technology could be used as a novel stabilization/solidification method for reverse osmosis reject waters, which frequently pose disposal problems.

## 1. Introduction

Global concrete production consumed 16.6 Gt of water in 2012, which was 18% of all industrial water consumption [1]. Of this amount, 2 Gt (or approximately 13%) were used as batching water (i.e., the water mixed with cement, fine and coarse aggregates, and admixtures) [1]. Batching water quality is not strictly specified in most concrete standards, but in practice, drinking-quality water is frequently used [2]. Moreover, high concrete demand and production coincides geographically with water-stressed areas where potable water has limited availability [1]. The use of drinking-quality water in concrete batching is not globally as severe sustainability issue as the high CO_2_ emissions of Portland cement production but it can be locally an important question.

Consequently, to mitigate the high water consumption of concrete manufacturing, various alternative water sources have been studied; for instance, secondary or tertiary-treated municipal wastewaters [3–5] or wastewater generated at ready-mix concrete plants [6, 7]. In coastal areas, sea water could provide a sustainable option for concrete batching. However, the use of sea water in regular Portland cement concrete causes accelerated setting and a risk of rapid corrosion of metal rebars due to high free chloride concentration [8]. In terms of strength development, most studies indicate that early age strength of concrete prepared with sea water increases and is unaffected in the long term in comparison to concrete prepared with potable water [9]. Hydration kinetics of Portland cement pastes prepared with increasing chloride concentration showed that the heat release associated with the dissolving period of the hydration process was diminished, and the hardening deceleration stage was postponed due to increased contact area for C_3_S, C_2_S, and C_3_AH phases [10]. Increased sodium chloride concentration also increases the dispersion of paste, as with superplasticizers [10]. In terms of microstructure, increasing chloride ion concentration up to 1.0% in Portland cement paste caused decreased porosity and subsequently also a decreased carbonation rate [11].

Reverse osmosis (RO) is a widely used high-pressure (≈ 10–90 bar) membrane process for the desalination of sea water, brackish water, and different kinds of wastewaters [12–16]. The estimated number of operational desalination plants worldwide was 18,000 in 2015 with a total capacity of more than 88 million m^3^/d [17]. A typical RO system produces concentrated reject water (or rejectate) 60–80% and 5–40% in the cases of sea water and brackish or low salinity feed waters, respectively [18]. RO reject waters are typically further concentrated and then disposed at sea, deep wells, evaporation ponds, or municipal sewers [19]. However, RO reject water disposal costs are typically high, they may pose technical challenges, and cause environmental issues [20, 21]. As one possible utilization prospect, RO rejectate was studied as batching water for mixtures of Portland cement and ground-granulated blast furnace slag (BFS): compressive strength was found to be practically unaffected, and chloride permeability decreased significantly indicating denser microstructure [22].

Alkali-activated materials (AAMs) and their subgroup geopolymers, are alternative Portland cement-free binders, which have been intensively studied and also utilized in construction projects to mitigate another environmental issue of concrete production: high CO_2_ emissions [23, 24]. However, the water demand for AAMs is approximately similar to Portland cement concrete; therefore, alternative water sources, such as sea water, have also been studied with AAMs [25–27]. With conventional (two-part) AAMs, water is added to the binder system in the form of alkali-activator solution (containing high concentration of, for instance, alkali hydroxide or silicate), whereas with one-part (i.e., just-add-water or dry mix) AAMs, water is added to a dry mixture of solid activator, aluminosilicate precursors, and aggregates [28]. Most studies concerning alternative water sources with AAMs focus on two-part systems, which behave differently in many aspects in comparison to one-part AAMs. In one of the few published studies, BFS and fly ash were activated with a mixture of powdered sodium hydroxide, silicate, and carbonate by the addition of sea water: accelerated setting, similar strength development as with tap water, and only limited steel rebar corrosion after one year were observed [29].

In the present work, simulated sea water and RO reject waters were studied for the preparation of one-part alkali-activated BFS mortar. The main research questions of the study were as follows: 1) how does the increased salt content affect the fresh and hardened properties, microstructure, and reaction kinetics in one-part alkali-activated BFS mortar, and 2) could alkali-activation technology be used for the solidification/stabilization of RO reject waters (i.e., to prevent the release of salts)? The hypothesis was that based on the existing literature of two-part alkali-activated systems prepared with high salinity water, the fresh and hardened properties should not deteriorate. However, little information about the stability of salts originating from batching water was available, and thus represents a knowledge gap. The solidification/stabilization efficiency was assessed with leaching tests for monolithic mortar prisms and crushed mortar to consider leaching of salt during in-service life and end-of-life scenarios, respectively.

## 2. Materials and methods

### 2.1. Materials

Precursor for alkali-activated mortar was ground-granulated BFS (Finnsementti, Finland) with d_10_, d_50_, and d_90_ values of 0.9, 10.8, and 51.7 μm, respectively. BFS had bulk and true densities of 1.20 and 2.93 g/cm^3^, respectively. The main components of BFS were (as oxides, w/w) CaO 38.51%, SiO_2_ 32.33%, MgO 10.24%, Al_2_O_3_ 9.58%, SO_3_ 4.00%, TiO_2_ 2.21%, and Fe_2_O_3_ 1.23%. Loss on ignition (LOI) was 0.46% (at 950°C). The solid activator was anhydrous sodium metasilicate (Alfa Aesar, Germany), with a silica modulus (molar SiO_2_/Na_2_O) of 0.9 and water content of 2.5%. Sand utilized in the mortar preparation was standard sand (Normensand, Germany) with a particle size ranging between 0.08 and 2 mm.

Substitute (or simulated) sea water was prepared according to the standard [30], which describes the average representative concentrations of salts in ocean water. Substitute sea water was employed instead of real sea water to have reproducible batching water throughout the experiments. RO reject water was prepared by considering a RO desalination process in which the water recovery was assumed to be 60% (i.e., the amount of pure water or permeate); and thus, the reject water was concentrated by a factor of 2.5. In short, simulated RO reject water was prepared by adding 2.5 times more salt than to the simulated sea water. The salt concentrations of the simulated sea water and RO reject water are shown in Table 1. Drinking-quality tap water (from Oulu, Finland) was utilized as a reference, and its chemical characteristics are shown in Table 2. Hardness of the tap water was 0.77 mmol/L.

**Table 1:** Concentrations of salts in simulated sea water and RO reject water (ASTM D1141–98, 2003).

Concentration [g/L]	Simulated sea water	Simulated RO reject water
NaCl	24.53	61.33
MgCl_2_	5.20	13.00
Na_2_SO_4_	4.09	10.23
CaCl_2_	1.16	2.90
KCl	0.695	1.738
NaHCO_3_	0.201	0.503
KBr	0.101	0.253
H_3_BO_3_	0.027	0.068
SrCl_2_	0.025	0.063
NaF	0.003	0.008

**Table 2:** Typical chemical characteristics of the tap water from Oulu, Finland.

Constituent [mg/L]	Tap water
Cl^-^	1.2
SO_4_^2-^	47.3
Total organic carbon (TOC)	1.5
Al	< 0.03
Ca	31.4
Fe	0.031
K	0.62
Mg	1.20
Mn	0.009
Na	2.34
P	< 0.05
S	14.8
Si	2.08

### 2.2. Preparation of mortars

The mortar mix design was as follows: sand/binder/water = 5.7/2.9/1.0 (weight ratio). The binder contained 90 wt% BFS and 10 wt% of solid anhydrous sodium metasilicate. The mortar was prepared by mixing dry solids for 3 min, adding water, and mixing for another 3 min. The mixture was cast in 40 × 40 × 160 mm^3^ prismatic molds and compacted using a jolting table. The specimens were placed in a curing chamber (22°C and 100% relative humidity) for approximately 24 h, demolded, and returned to the curing chamber until testing.

### 2.3. Microstructure and chemical characterization

The chemical composition of BFS was determined using a 4 kV wavelength dispersive X-ray fluorescence (XRF) spectrometer (PANalytical AxiosmAX). XRF analyses were performed from the fused samples: 1.5 g of the sample was melted at 1150°C with 7.5 g of X-ray Flux Type 66:34 (66 wt% Li_2_B_4_O_7_ and 34 wt% LiBO_2_) to obtain melt-fused tablets. The particle size distribution of BFS was determined using the Beckman Coulter LS 13320 laser diffraction particle size analyzer. The loss on ignition (LOI) of BFS was found by determining a mass decrease of a constant weight sample (dried at 105°C for 24 h) after heating to 950°C.

Isothermal calorimetry experiments were conducted using a TAM Air isothermal calorimeter and 20 mL glass admix ampoules with a stirrer (TA Instruments) at a temperature of 25 ± 0.01°C. BFS, sodium silicate, and sand were loaded into the ampoule while water (tap, sea, or RO reject water) was loaded into two 1-mL syringes. An ampoule containing deionized water was used as a reference. Materials were equilibrated for 2 to 4 h inside the calorimeter chamber and then mixed for 3 min using an up and down motion with the stirrer. The heat release rate values were normalized by the mass of the binder (i.e., BFS and sodium silicate).

A field emission scanning electron microscope with an energy dispersive spectroscope (FESEM-EDS, Zeiss Ultra Plus) was used to take micrographs of the hardened mortar intersections and to provide semi-quantitative information about chemical composition. Analyses were conducted using a backscatter electron detector with a 15 kV acceleration voltage and an 8.5 mm working distance. The samples were polished with P120, P240, and P1200 abrasive grinding papers and ethanol flushing to reveal cross-sections. The samples were coated with carbon prior to measurement.

Crystalline phases were identified and quantified using X-ray diffraction (XRD) with a Rigaku Smartlab diffractometer (9 kW Cu X-ray source) in the range of 5 to 120°2θ using 6°2θ/min scan speed. XRD analyses were performed from paste samples at 28 days of age. Before analyses, the samples were pulverized, and 10 wt% of rutile (≥ 99.9%; Aldrich, Germany) was added as an internal standard. Quantification of phases was performed using the Rietveld refinement method, which also allows estimation of the amorphous content.

### 2.4. Fresh and hardened state properties characterization

The initial and final setting times were determined with a Vicat apparatus (Vicatronic Matest) from paste samples according to a standard method [31]. Workability was evaluated from mortar samples using the flow table test according to a standard method [32].

The compressive and flexural strength developments were determined with a Toni Technik 3000 kN testing machine. Initially, the prismatic beams were assessed under flexural loading, and the broken half-specimens were used for the compressive strength measurement. Loading rates were 50 N/s and 2.4 kN/s during the flexural and compressive strength tests, respectively, according to the standard [33].

Porosity was assessed using the Archimedes method [34]. Dried samples (at 60°C for 1 week) with a known mass were exposed for vacuum (p ≈ 5 kPa) for 2 h, submerged in deionized water, and kept immersed under water for 24 h. The mass of samples was recorded as submerged in water and as water-saturated in air. Total porosity was calculated according to Eq 1 (m_s_ = weight in saturated condition in air [g], m_d_ = dry sample weight [g], m_h_ = sample weight suspended in water [g], ρ_r_ = true density [g/cm^3^], ρ_rh_ = density of water [g/cm^3^]). True density was measured from a pulverized sample with a He gas pycnometer (AccuPyc II 1340, Micromeritics).

$$Total\;porosity\;[\%]=(1-\frac{\frac{m_{d}}{m_{s}-m_{h}}\times \rho_{rh}}{\rho_{r}})\times 100$$ (1)

### 2.5. Efflorescence and leaching of salts

Efflorescence formation was evaluated by immersing one day old mortar samples in tap water so that half of the specimen was submerged in water and half was exposed to air. The formation of efflorescence was monitored for up to 28 days.

The leaching of salts from crushed mortars was evaluated by applying a standard two-stage batch leaching test [35]. In short, mortars (at 28 d age) prepared with tap, sea, or RO reject water were crushed to particle size of less than 4 mm and extracted with distilled water at the liquid/solid (L/S) mass ratio of 2 for 6 h followed by a similar extraction at the L/S ratio of 8. The mass of crushed mortar material was 87.5 g. Eluates from both steps were filtered with 0.45 μm membrane filter and analyzed for total concentrations of Na and Ca (with an inductively coupled plasma optical emission spectrometer, Thermo Fisher Scientific iCAP6500 Duo) and anions chloride, sulfate, bromide, and fluoride (with an ion chromatography system, Dionex, ICS-2000). The leached amount per dry mass in the first and second leaching steps were calculated with Eqs 2 and 3, respectively (A_2_ = the release of a constituent at a L/S = 2 [mg/kg]; C_2_ = the concentration of a constituent in the eluate [mg/L]; L_2_ = the volume of leachant [L]; MC = the moisture percentage of the dry mass; A_2-10_ = the cumulative release at a cumulative L/S = 10 [mg/kg]; C_8_ = the concentration of a constituent in the eluate from the second extraction [mg/L]; L_8_ = the volume of leachant in the second extraction [L]; V_1_ = the volume of eluate from the first extraction [L]).

$$A_{2}=C_{2}(\frac{L_{2}}{M_{D}}+\frac{MC}{100})$$ (2)

$$A_{2-10}=C_{2}\frac{V_{1}}{M_{D}}+C_{8}(\frac{L_{2}+L_{8}-V_{1}}{M_{D}}+\frac{MC}{100})$$ (3)

Another leaching test was conducted by applying a standard method for monolithic building materials [36]. In this test, prismatic mortar beams (40 × 40 × 160 mm^3^) prepared with tap, sea, or RO reject water were exposed to deionized water acidified to pH 4 with nitric acid at the liquid/mortar beam volume (L/V) ratio of 8. Containers were sealed, allowed to stand still at room temperature, and acidified water was replaced at 6 h, 24 h, 5 d, 16 d, 36 d, and 64 d. Eluates were analyzed for total concentration of Ca and Na and for anions chloride, sulfate, and bromide. The following parameters were calculated: the measured leaching of a component in fraction ($E_{i}^{*}$, mg/m^2^), the cumulative leaching ($\varepsilon_{n}^{*}$, mg/m^2^), and the derived cumulative leaching (ε_n_, mg/m^2^) according to Eq 4 to 6, respectively (c_i_ = the concentration of the component [mg/L]; V = the volume of eluate [1.28 L]; A = the surface area of prismatic beams [0.0288 m^2^]; t_i_ = the time at the end of fraction [s]; and t_i-1_ = the time at the start of the fraction [s]).

$$E_{i}^{*}=\frac{c_{i}\times V}{A}$$ (4)

$$\varepsilon_{n}^{*}=\sum_{i=1}^{n}E_{i}^{*}$$ (5)

$$\varepsilon_{n}=(E_{i}^{*}\times \sqrt{t_{i}})/(\sqrt{t_{i}}-\sqrt{t_{i-1}})$$ (6)

## 3. Results and discussion

### 3.1. Isothermal calorimetry

**Fig 1:**
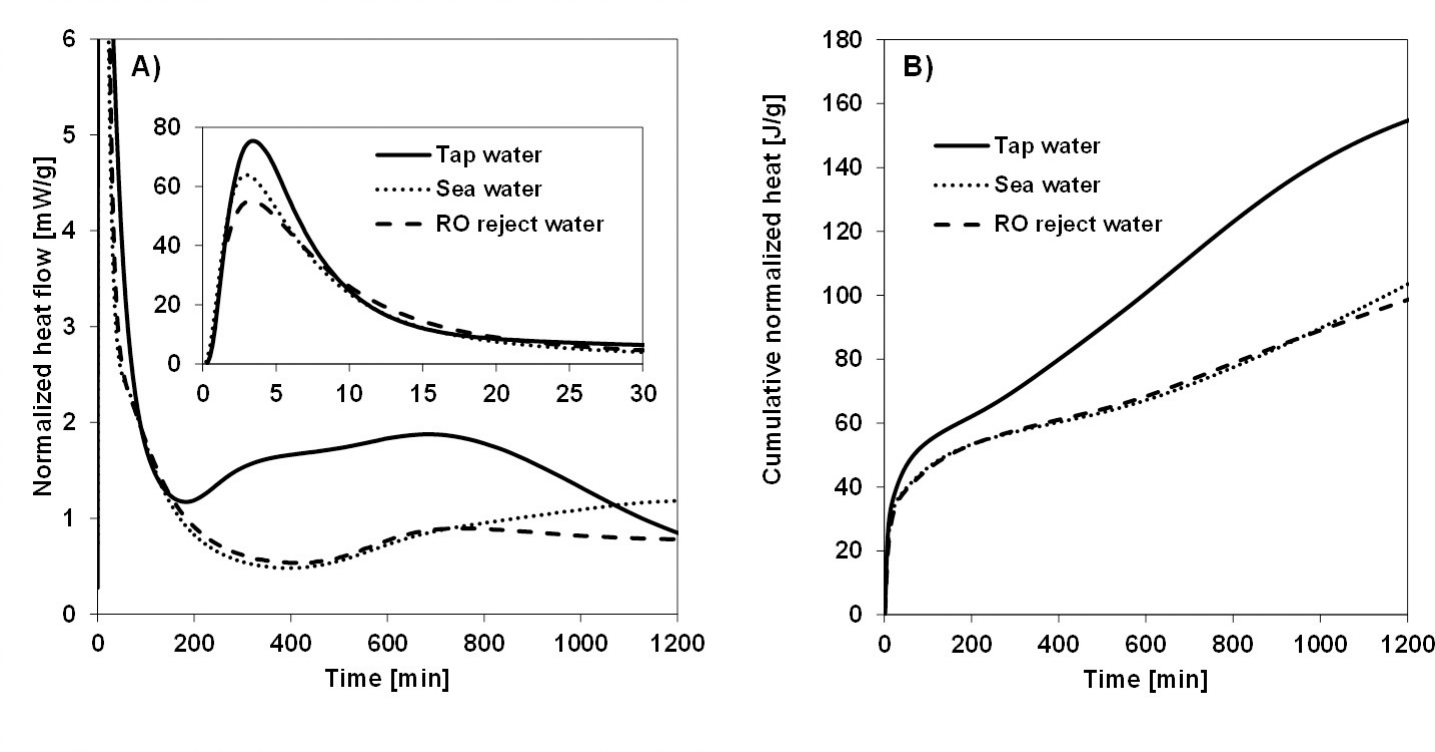
Heat generation curves of mortars prepared with different batching waters. A) normalized heat flow and B) cumulative normalized heat as measured by an isothermal calorimeter.

**Fig 2:**
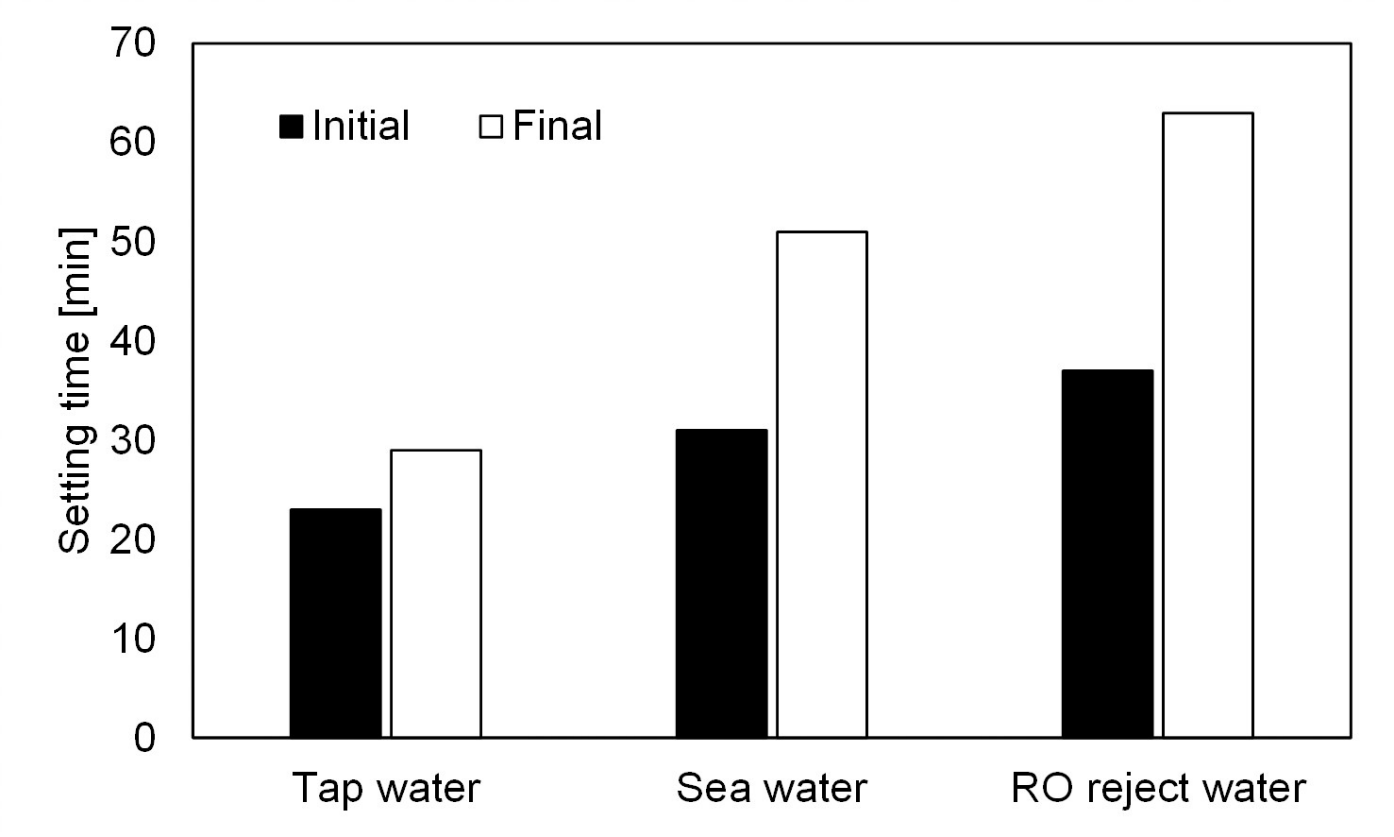
Initial and final setting times of pastes prepared with different batching waters.

Normalized heat flow curves (Fig 1A) of mortars prepared with different batching waters contained an initial peak (wetting and dissolution of solid activator), a short induction period (build-up of dissolved silica and alumina), and a second peak (nucleation, growth, and precipitation of (C,N)-(A)-S-H gel) [37–40]. The intensity of the initial peak decreased as the salinity of batching water increased. This may reflect the decreased dissolution of sodium silicate as the concentration of salts increased in the water (i.e., the salting-out effect of silica, which is discussed more in Section 3.2). The induction period, which was not very well noticeable, ended after approximately 200 min in the case of tap water and approximately 400 min in the case of sea or RO reject waters. These findings were also supported by setting time measurements (Fig 2). The second peak started to diminish after 800 min when using tap water, whereas with more saline waters, it was still growing at 1200 min. The cumulative normalized heat (Fig 5B) indicated that the use of tap water resulted in approximately 57% higher heat generation in comparison to saline waters after the first 20 h. Moreover, the increased salinity (from sea water to RO reject water) did not further affect the cumulative heat release during this period. Since the heat release was approximately 30% lower than with Portland cement within the same time-frame [41], the one-part AAMs prepared with high-salinity batching water could be used in applications requiring low-heat binders.

### 3.2. Setting time and workability

Initial and final setting times increased as the salinity of batching water increased (Fig 2). NaCl (the main salt present in the studied saline waters) has been reported to decrease the setting time of two-part alkali-activated BFS at doses < 4% (of weight of slag), whereas higher concentrations increased setting time [42]. The suggested mechanisms included changes in the rate of gelation and hydration [42]. In the present study, the amount of NaCl was approximately 1% of slag, which indicated that the mechanism affecting setting would differ from that of two-part mix designs. In addition, contradictory results to the present study were also obtained from another work involving one-part AAM: sea water was reported to decrease the setting time of one-part BFS/fly ash binder activated with a mixture of powdered sodium silicate, hydroxide, and carbonate in comparison to tap water [29]. Thus, the literature indicates that the selection of precursor and activator affects the trend in setting time (i.e., increases or decreases) when using saline batching water. For instance, even small changes in the BFS composition can have drastic effects on the reaction chemistry [43, 44]. An explanation for the observed increasing setting time in the present study could be due to the salting-out effect of silicate in high-salinity water [45]. In this context, the salting-out effect means, that the solubility of sodium silicate is reduced due to interactions of salt ions with water molecules in saline batching water. In fact, a decreased silica solubility was observed when the concentration of aqueous NaCl exceeded 0.5 M in one study [46]. In the present study, the total concentration of salts was 0.53 M in sea water and 1.32 M in RO reject water. Workability of the mortars was unaffected by the increase of salinity in batching water: the differences in flow table spread values with tap, sea, and RO reject waters (217–221 mm) were all well within the margin of error.

### 3.3. Compressive strength

**Fig 3:**
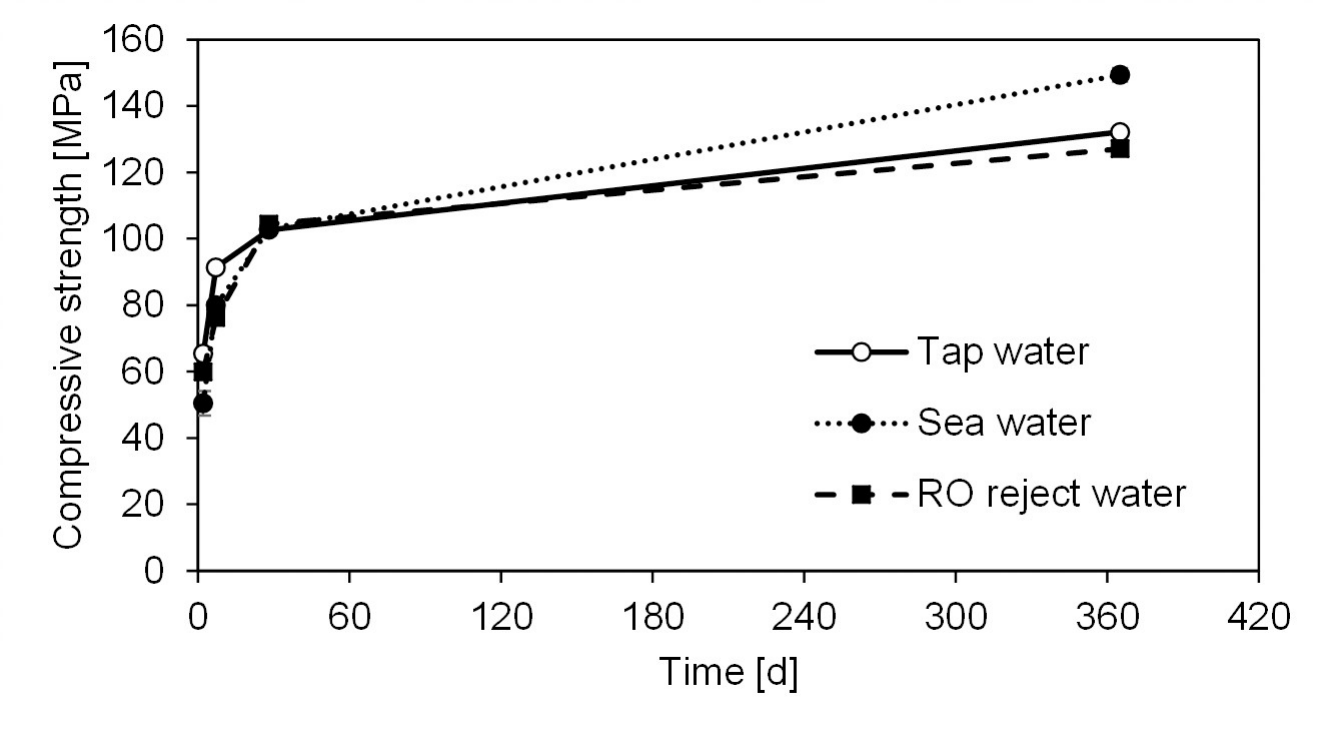
Compressive strength of mortars prepared with different batching waters. Each value is an average of six measurement and error bars represent a ±1 standard error.

Compressive strength of the mortars (Fig 3) prepared with simulated sea water or RO reject water was approximately 8% to 17% lower in comparison to tap water at 2 or 7 d age. At 28 d age, the differences between the three mortars were statistically nonsignificant according to the one-way analysis of variance (ANOVA, α = 0.05, p = 0.12). At the final age of 365 d, the strength obtained with sea water was approximately 13% and 17% higher than with tap water or RO reject water, respectively. The improved strength when using higher salinity batching water could be due to less porous structure of mortar (see section 3.4). The strength development trend differed from Portland cement-based systems [9] or two-part AAM system (BFS activated by a solution of sodium silicate and hydroxide) [27] in which the early strength was higher with saline bathing water than with fresh water. It has been suggested that this enhanced early strength development occurred in a manner that was similar to CaCl_2_ setting accelerator: a rapid diffusion of Cl^-^ through the initially formed C_3_S hydrates causing faster diffusion of other ions and hydration reactions possibly due to more permeable C-S-H layers [47]. The present mix design resembled the one used by Yang et al. (2019), with the exception that BFS was activated by solid sodium silicate activator instead of a solution. Therefore, one possible explanation for the slightly lower compressive strength at early ages could again be the hindered dissolution of the solid sodium silicate activator in high-salinity water.

### 3.4. Microstructure

**Fig 4:**
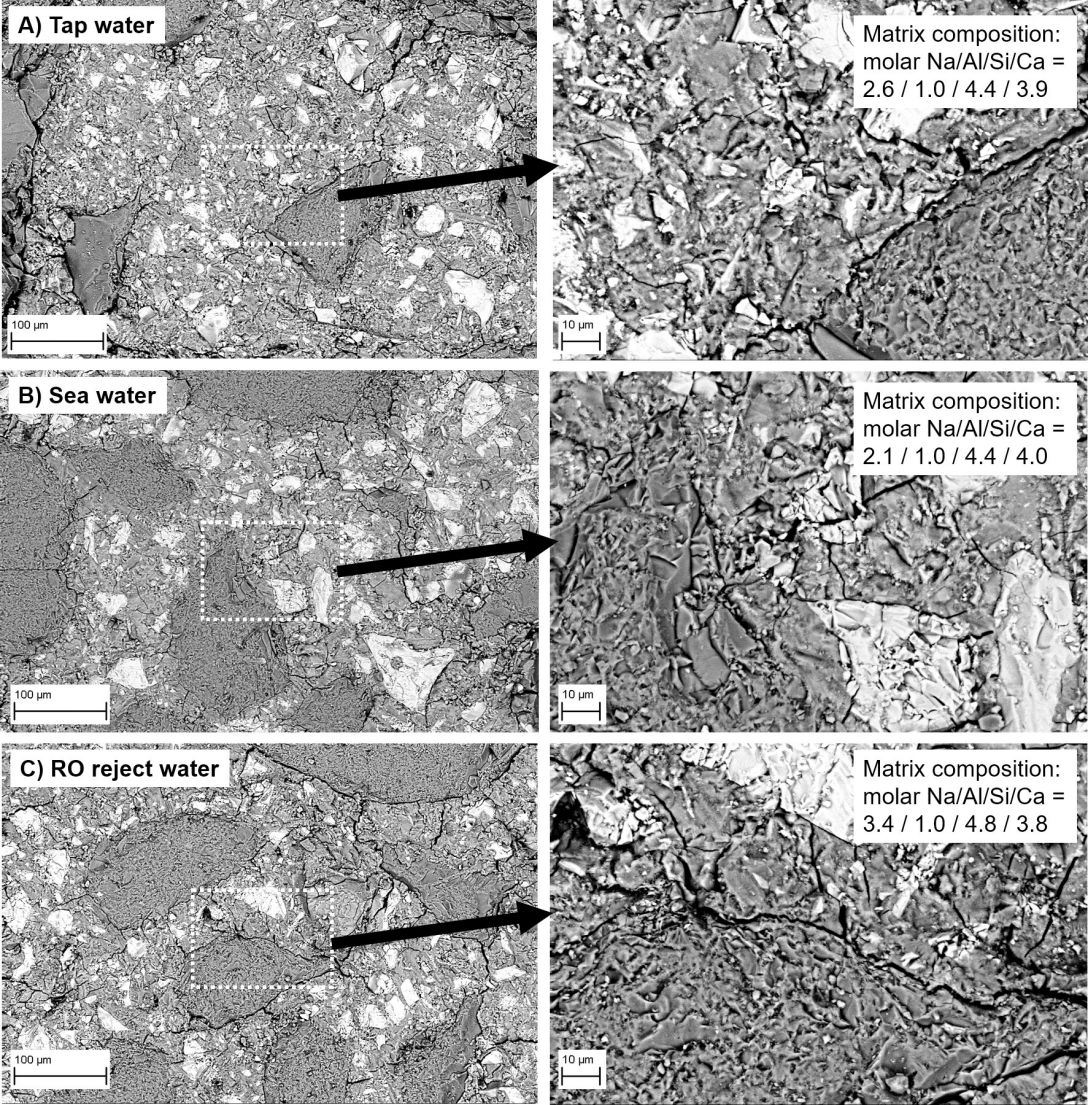
Micrographs of mortar intersections and matrix molar compositions (as determined by X-ray microanalysis).

Micrographs (Fig 4) revealed that the microstructure of mortars prepared with different batching waters was similar in terms of aggregate-mortar interface and the amount of unreacted BFS. The porosity of mortars decreased slightly as the water salinity increased: total porosities (as determined by the Archimedes method) when using tap, sea or RO reject water were 9.1, 8.4, and 8.2%, respectively. This indicated that the increasing salt content could fill some of the pore volume. Furthermore, the average binder matrix composition (Fig 4) was very similar between tap water- and sea water-based mortars. However, when RO reject water was used, slightly elevated Na and Si amounts could be observed.

**Fig 5:**
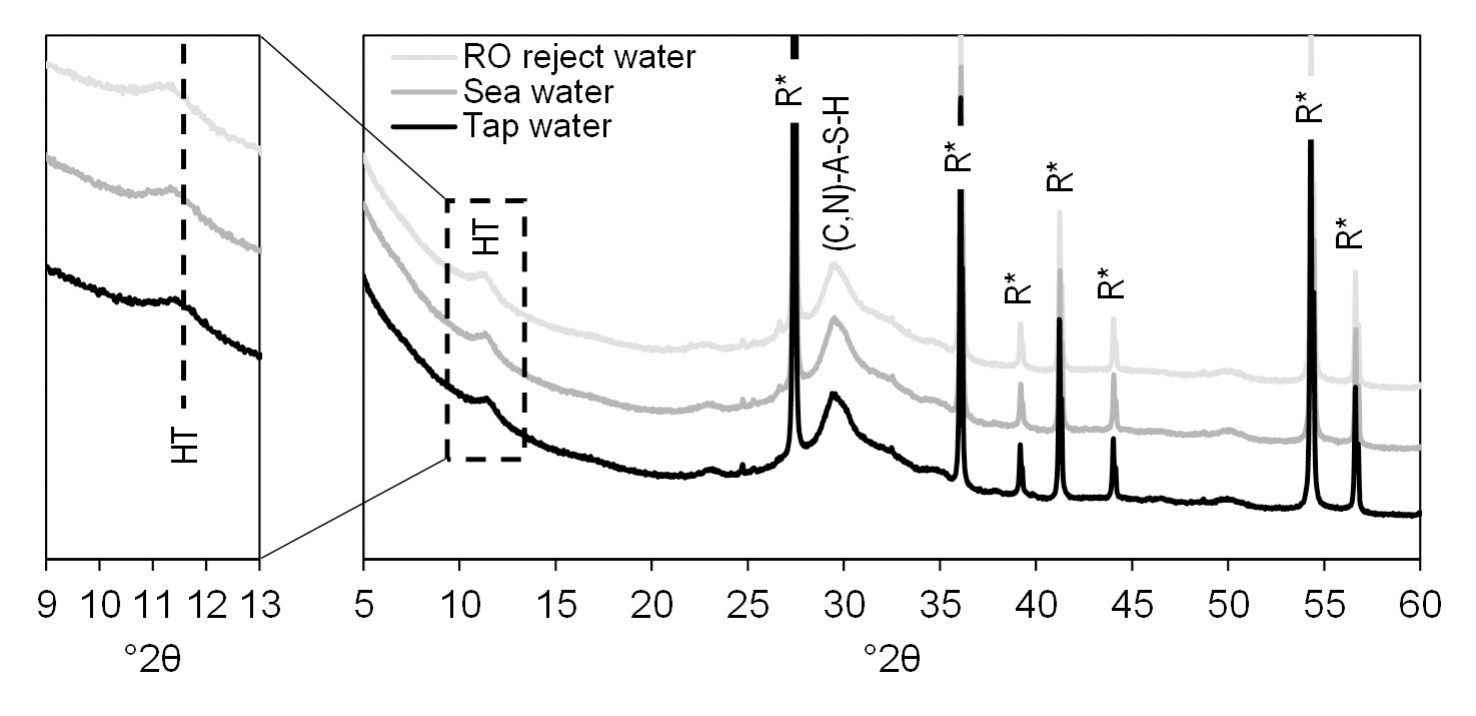
Diffractograms of pastes at 28 d age prepared with tap water, sea water, and RO reject water. HT = hydrotalcite-like phases and R* = rutile, internal standard (TiO_2_). The HT peak is shifting due ion-exchange of chloride into the phase (in the zoomed part of the figure).

Diffractograms of the paste samples prepared with different batching waters (at 28 d age) are shown in Fig 5. The Rietveld analysis (with the addition of rutile as an internal standard) revealed that the samples were completely amorphous or nano-crystalline. However, there was a broad peak at approximately 12°2θ in all samples, indicating the presence of weakly ordered hydrotalcite-like phases (M_x_^2+^M_y_^3+^(OH)_2x+3y−nz_(A^n−^)_z_·mH_2_O, in which M_x_^2+^, M_y_^3+^, and A^n−^ are Mg^2+^, Al^3+^, and CO_3_^2−^, respectively, and x/y is 2–3 for alkali-activated BFS prepared with freshwater [48–50]). The low structural ordering of the formed hydrotalcites is typical when BFS is treated with sodium silicate activator [48, 51, 52]. The hydrotalcite peak position (Fig 5) appeared to shift slightly when the salinity of batching water increased. This indicated that chloride ion exchange may take place in the hydrotalcite phase, which resulted in layer spacing increasing (from approximately 7.7 Å to 8.0 Å) and subsequently shifting of the peak position [53]. In addition, the broad peak of the (C,N)-A-S-H gel at approximately 30°2θ was very similar between samples. However, the similarity of the paste samples was to be expected as the precursor and activator were similar in all cases. It appeared that the concentrations of salts in batching waters were too low to form new phases detectable by XRD.

### 3.5. Efflorescence and leaching of salts

**Fig 6:**
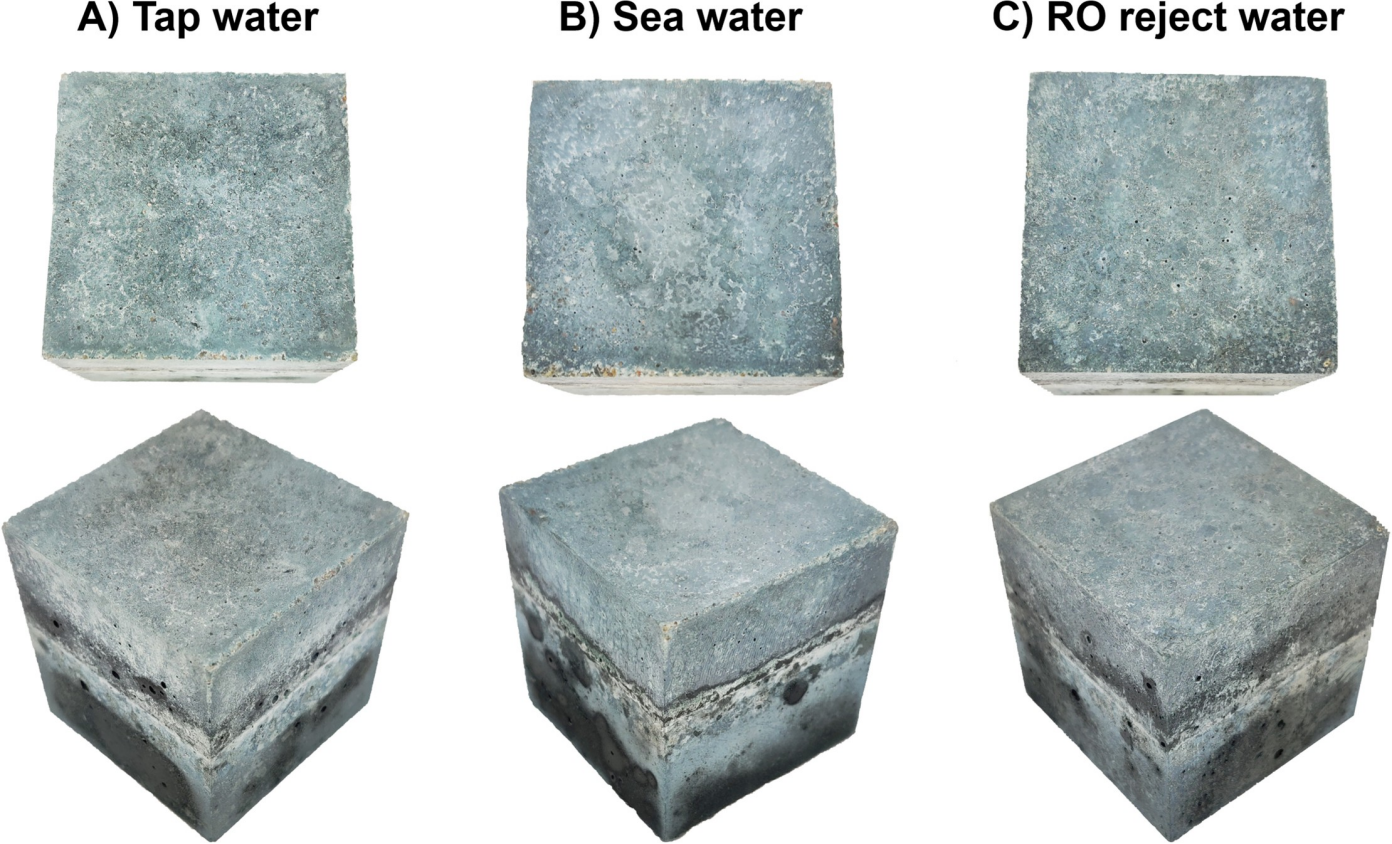
Visual examination of mortar samples (50 × 50 × 50 mm^3^ cubes) after they were half submerged in deionized water for 28 days: No formation of efflorescence on the top surface.

Mortar samples did not develop any visible efflorescence when half-exposed to deionized water for 28 d (Fig 6). This indicated that the salts contained in the binder matrix were unable to migrate to the surface of the mortar due to capillary action, and thus were chemically and/or physically stabilized into the structure.

**Fig 7:**
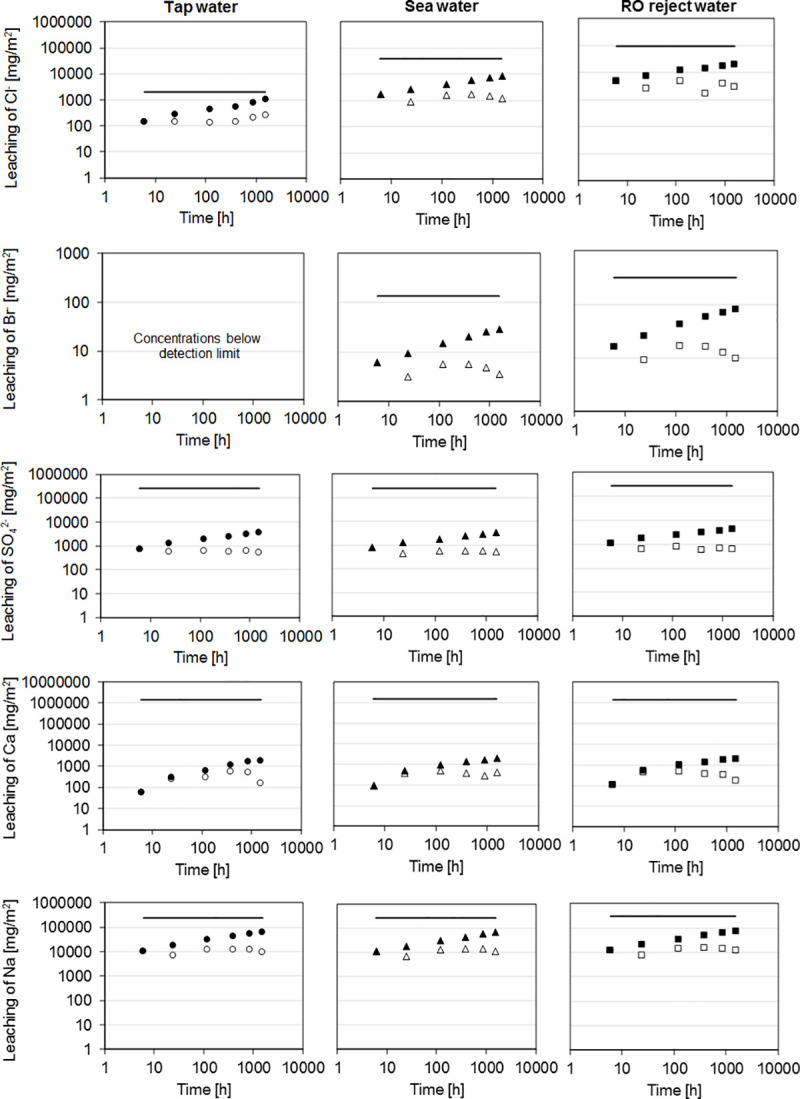
Results of diffusion leaching tests for mortars prepared with tap water (in the left), sea water (in the middle), and RO reject water (in the right). The white circles, triangles, and squares refer to leaching at the point of time, whereas the black ones refer to cumulative leaching. The horizontal line refers to the total amount of the component.

The stability of salts contained in the mortar prisms was quantitatively assessed with the diffusion test for monolithic building materials, that is, by submerging the samples in water at initial pH of 4 [36]. In general, the leaching of different components (Fig 7) was controlled by dissolution (or wash-out) rather than diffusion since the slopes at different parts of log ε vs log t plots were < 0.65 [36]. In addition, it is typical for this kind of behavior that the dissolution per interval does not follow the cumulative dissolution [54]. The dissolution-controlled mechanism means that the dissolution of surface material is faster than the diffusion through the pore structure of the material [54]. Thus, the observed leaching was due to the surface of monoliths releasing the salts. When the leaching test was conducted for crushed mortar, the amount of released components increased (Table 3), which was due to higher exposed surface areas. The study with crushed mortar represents an end-of-life scenario. Therefore, the results were compared to corresponding waste material guideline values.

**Table 3:** Results of two-stage leaching tests for crushed mortars (to evaluate end-of-life behavior) prepared using tap water, sea water, and RO reject water. Results are compared to the requirements of the European Council Decision 2003/33/EC [60].

	Tap water	Sea water	RO reject water	Requirements per the European Council Decision 2003/33/EC	
	First stage (L/S = 2)			Inert waste	Non-hazardous waste
Cl^-^ [mg/kg]	20	1748	3180	550	10000
Br^-^ [mg/kg]	42	6	32	-	-
SO_4_^2-^ [mg/kg]	458	724	528	560	10000
Ca [mg/kg]	13	14	15	-	-
Na [mg/kg]	5120	6380	6560	-	-
pH	12.3	12.5	12.7	> 6	> 6
Conductivity [mS/cm]	15.6	17.7	19.3	-	-
	Second stage (cumulative L/S = 10)			Inert waste	Non-hazardous waste
Cl^-^ [mg/kg]	55	1726	3483	800	15000
Br^-^ [mg/kg]	67	6	51	-	-
SO_4_^2-^ [mg/kg]	660	916	874	1000	20000
Na [mg/kg]	8464	9618	9951	-	-
Ca [mg/kg]	99	120	148	-	-
pH	12.1	12.1	12.1	> 6	> 6
Conductivity [mS/cm]	5.3	5.3	5.5	-	-

For chloride, the cumulative leaching from monolithic samples increased by a factor of 8.5 and 20.7 with sea water and RO rejectate, respectively, in comparison to tap water. The maximum chloride concentrations in leachate of monolithic samples were approximately 40 and 114 mg/L in the case of sea water and RO rejectate, respectively. To put these numbers into perspective, the threshold concentration for taste in water is 250–300 mg Cl^-^/L [55] and the US EPA recommends a maximum concentration in drinking water of 250 mg Cl^-^/L [56]. Since there was no detected Friedel’s salt (Ca_2_Al(OH)_6_(Cl,OH)·2H_2_O) or Kuzel's salt (Ca_4_Al_2_(SO_4_)_0.5_Cl(OH)_12_∙6H_2_O), which are frequently sinks of chloride in cementitious systems [57], chloride was likely retained by adsorption or ion-exchange to the hydrotalcite-like phases [58] and C-(A)-S-H gels [59]. The ion-exchange on hydrotalcite was supported by the corresponding peak shifting in the diffractogram as salinity increases (see Fig 5). For crushed mortar, the two-stage leaching of chloride exceeded the landfilling guideline values for inert waste but were clearly within those for non-hazardous waste. This should be taken into account when planning possible reuse of the mortar or concrete after its lifetime.

Bromide leaching from monolithic samples prepared using sea water or RO rejectate was minimal, and the observed maximum concentrations were only 0.14 and 0.38 mg Br^-^/L, respectively. These values were lower than those typically encountered in fresh water bodies [55]. Bromide release in the two-stage leaching test exhibited no clear trend between the different bathing waters: for example, mortars prepared from sea water resulted in the lowest release, and tap water-based mortar released more bromide than RO rejectate-based mortar in the second step. Nevertheless, bromide is not regulated in the European Council Decision 2003/33/EC [60].

Sulfate leaching from monolithic samples (Fig 7) was approximately similar regardless of the batching waters used even though the concentrations varied notably. There was approximately 47, 2800, and 6900 mg SO_4_^2-^/L in tap, sea, and RO reject waters, respectively. The maximum concentration of sulfate in the leachate of monolithic samples was 25.9 mg/L, which is well below the drinking water guideline (250 mg/L) [55]. This indicated efficient stabilization. In fact, alkali sulfates can be used as alternative activators for high-calcium precursors [24]. The sinks of sulfate in these systems are ettringite (Ca_6_Al_2_(SO_4_)_3_(OH)_12_·26H_2_O), AFm, and AFm (calcium mono- and trisulfoaluminates, respectively) [61]. However, none of these phases was detected with XRD (Fig 5), which could be due to their relatively low amounts in the binder. In two-phase leaching test, the sulfate release was above the inert waste limit in the first phase in the case of sea water. In the second phase, the values were below the limit values.

Leaching of calcium in the diffusion test (Fig 7) exhibited similar behavior for all batching waters and the maximum detected concentration was only 11.9 mg/L. Calcium is stably incorporated in C-(A)-S-H gel; therefore, its release is limited. However, in the second stage of the two-stage leaching test, calcium release increased as a function of salinity but still the concentration remained only up to 120 mg/L.

Sodium leaching from monoliths was approximately similar for all batching water. The maximum sodium concentration was 353 mg/L, which is above the threshold that can cause taste in drinking-water guideline (200 mg/L) [55]. However, there is no health-based guideline for sodium content in drinking water [55]. Sodium is not also not regulated in the European Council Decision 2003/33/EC [60].

The eluents from two-stage leaching test (Table 3) exhibited an increasing trend in terms of pH as salinity increased. In the second stage, however, pH was constant for all crushed mortars. According to the requirements of 2003/33/EC [60], pH should be over 6. Conductivity increased slightly in the first stage of the two-stage leaching test and was approximately constant in the second phase. This behavior was likely connected to the pH of the effluents as OH^-^ is a major anion affecting conductivity: its molar ionic conductivity is 198.6 (S·cm^2^)/mol).

## 4. Conclusions

This work provided a proof-of-concept study relating to utilizing high-salinity batching waters for the preparation of one-part alkali-activated blast furnace slag mortar. The results demonstrated that the heat release upon adding water decreased as the salinity of batching water increased. At the same time, the setting time of mortars increased. These trends are possibly linked to the salting-out phenomenon of silicate from solid activator, which has been documented in the literature. Compressive strength development was slightly lower with high-salinity batching waters at early age, but higher final strength is obtained in comparison to tap water. This could be explained by the slightly lower porosity of mortars prepared with saline waters. The microstructure of the studied mortars was approximately similar based on micrographs, X-ray microanalysis, and X-ray diffraction. The mortars exhibited generally good stability in terms of salt leaching.

The results provide useful prospects for safe disposal of reverse osmosis reject water, which at the moment poses great challenges for desalination plants and mine water treatment facilities. Reject water could be solidified/stabilized in alkali-activated calcium aluminosilicate matrix and the obtained mortar or concrete could be utilized for construction purposes. Leaching of salts is minimal when the material is in the form of a monolith. For the end-of-life considerations of binders, crushed mortars were also studied. In that case, the material met the minimum standards for non-hazardous waste.
